# Single nucleotide polymorphisms for parentage testing of horse breeds in Korea

**DOI:** 10.5713/ab.23.0123

**Published:** 2023-10-27

**Authors:** Sun-Young Lee, Su-Min Kim, Baatartsogt Oyungerel, Gil-Jae Cho

**Affiliations:** 1 Racing Laboratory, Korea Racing Authority, Gwacheon 13822, Korea; 2 Department of Horse Industry, Sung Woon University, Yeongcheon 38801, Korea; 3 School of Animal Science and Biotechnology, Mongolian University of Life Sciences, Ulaanbaatar17024, Mongolia; 4 College of Veterinary Medicine, Kyungpook National University, Daegu 41566, Korea

**Keywords:** Horse, Microsatellite Marker, Parentage Testing, Single Nucleotide Polymorphisms, South Korea

## Abstract

**Objective:**

In this study, we aimed to evaluate the usability single nucleotide polymorphisms (SNPs) for parentage testing of horse breeds in Korea.

**Methods:**

The genotypes of 93 horse samples (38 Thoroughbred horses, 17 Jeju horses, 20 Quarter horses, and 18 American miniature horses) were determined using 15 microsatellite (Ms) markers (AHT4, AHT5, ASB2, ASB17, ASB23, CA425, HMS1, HMS2, HMS3, HMS6, HMS7, HTG4, HTG10, LEX3, and VHL20) and 101 SNP markers.

**Results:**

Paternity tests were performed using 15 Ms markers and 101 SNP markers in Thoroughbred horses and Quarter horses. AHT5, ASB2, ASB17, ASB23, CA425, HMS7, HTG10, and LEX3 did not follow Mendelian inheritance in Thoroughbred horses, whereas in Quarter horses, only AHT4, ASB2, and HMS2 showed Mendelian inheritance, consequently, paternity was not established. Meanwhile, 31 markers, including MNEc_2_2_2_98568918_BIEC2_502451, in Thoroughbred horses, and 30 markers, including MNEc_2_30_7430735_BIEC2_816793, in Quarter horses did not conform with Mendelian inheritance and therefore, could not be used for establishing parentage.

**Conclusion:**

The possibility of replacing Ms markers with SNP markers for paternity testing in horses was confirmed. However, further research using more samples is necessary.

## INTRODUCTION

The registration of Thoroughbred horses in Korea is implemented in accordance with the International Agreement on Breeding and Racing and the domestic Thoroughbred Horse Registration Regulations. Individual identification and parentage verification for registration of Thoroughbred horses had been conducted using blood-typing tests for nearly 40 years. However, blood-typing tests have the following disadvantages: i) they require fresh whole blood for testing, however, the cost of blood collection by veterinarians and transportation to the laboratory are high; ii) labor costs are high because of the long testing time and the manual nature of the tests; and iii) the accuracy of the results is low, at 97.0% to 99.5%. Because of these reasons, in the early 2000s, microsatellite (Ms) DNA typing was introduced to replace blood-typing tests in Korea for horse parentage testing, and its use has been continued till present day. For Ms testing, semiautomatic tests of blood or hair roots samples can be performed using analytical equipment, with approximately 99.99% accuracy of results [1].

Recent, research in many countries has focused on the introduction of the single nucleotide polymorphism (SNP) method, to replace Ms in horse registration [2,3].

According to the appropriate number and selection of SNP markers, SNPs genotypes can be used for estimating genomic breeding values for multiple traits [4], population monitoring and diversity management [5], and parentage control [6]. Compared with Ms markers, SNPs have a low mutation rate and afford a clear standardization of alleles, as reviewed by Vignal et al [7]. Concomitantly, it facilitates the laboratory automation of the analytical process, which reduces costs and enables a higher throughput in the generation of SNP genotype data [7,8].

Against this background, in this study, SNPs were analyzed and compared with the Ms results with the aim of securing basic data for future horse registration based on SNPs.

## MATERIALS AND METHODS

### Ethical statement

This study was a routine test for the registration of Thoroughbred horses in Korea and was not approved by the Research Ethical Committee, however, in this study, sampling was performed according to the guidelines for the care and use of experimental animals of the Animal Ethics Committee of the Korea Racing Authority.

### Sample collection and DNA extraction

Genomic DNA was obtained from 93 horse hair roots samples (38 Thoroughbred horses, 17 Jeju horses, 20 Quarter horses, and 18 American miniature horses) using an automated extraction system with a Nimbus KingFisher Presto instrument (Thermo Fisher Scientific, Inc, Waltham, MA, USA), according to the manufacturer’s protocols [9]. These horses are four genetically independent breeds (light breed and pony breed). The Thoroughbred horses used in this study for parental judgment were referred to the sample of horses registered in South Korea. For the concentration and purification of the sample, 1.2 μL of the extracted DNA was measured using an Epoch microplate spectrophotometer and the Gen5 3.00 program (Agilent Technology, Santa Clara, CA, USA). Samples that yielded a single band near 23 kb on electrophoresis (0.5× Tris-acetate-ethylenediaminetetraacetic acid, 1% agarose gel, 100 V, 30 min) with a DNA concentration of 10 ng/μL or more and an A260/A280 ration of 1.5 or more were deemed suitable for analysis.

### Single nucleotide polymorphism analysis

Quality control (QC) and quality assurance of genotype data for genome-wide association studies were performed according to the method reported by Laurie et al [10].

SNP analysis was performed in four stages (DNA amplification, DNA fragmentation, DNA resuspension and hybridization, and ligate-wash image scanning), followed by analysis using the Axiom Equine genotype sequence (Axiom MNEC670; ThermoFisher Scientific GeneTitan MC Instrument, USA).

### Microsatellite marker analysis

A total of 15 microsatellite loci (AHT4, AHT5, ASB2, ASB17, ASB23, CA425, HMS1, HMS2, HMS3, HMS6, HMS7, HTG4, HTG10, LEX3, and VHL20) were used for the analysis of the horses. Polymerase chain reaction (PCR) was performed according to the manufacturer’s protocols (Equine Genotypes Panel 1.1 Kit; ThermoFisher Scientific, USA). PCR products were tested using an automatic genetic analyzer (AB 3500XL Genetic Analyzer; ThermoFisher Scientific, USA); subsequent electrophoresis was performed on a POP 7 polymer (ThermoFisher Scientific, USA) at 15 kV. Using peak row data, the size of the alleles (in base pairs) of each marker was determined based on the results of the 2017/2018 Horse Comparison Test No. 1 of the International Society for Animal Genetics (ISAG), using 3500XL Data Collection ver. 5.0 and GeneMapper Software ver. 5.0 (ThermoFisher Scientific, USA). Alleles were showed using alphabetical symbols, in the order of smallest to largest, based on the assignment of a middle-sized allele as M. These definitions were confirmed by the International of Society and Animal Genetics (ISAG) horse comparison test.

### Statistical analysis and parentage testing

The SNP call rate, Fisher’s linear discriminant (FLD), heterozygous strength offset (HetSO), and homozygote ratio offset (HomRO) were analyzed according to the manufacturer’s protocols [11]. The SNP minor allele frequency (MAF) values were analyzed according to the method reported by Plzhnikov et al [12]. A shift in the MAFs can reflect mis-clustering events over the samples on the plates used. Chi-squared analysis is a simple method for automatically detecting this type of effect [12]. The allele frequencies of each SNPs and Ms markers were determined by direct counting from the tests performed for the 93 horses. Based on the SNPs and Ms markers results obtained in this study, we compared parentage testing using SNP and Ms in the same Thoroughbred horses and Quarter horses.

## RESULTS

### Quality control and single nucleotide polymorphisms analysis

**Figure 1:**
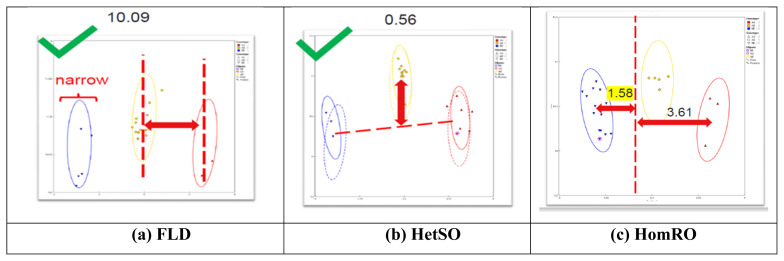
Results of the analysis of single nucleotide polymorphisms (SNPs) QC parameters (FLD, HetSO, and HomRO). (a) Fishers linear discriminant (FLD): measurement of the cluster quality of a SNP. High-quality SNP clusters exhibit: i) well-separated centers, and ii) narrow clusters. Default ≥ 3.6. (b) Heterozygous strength offset (HetSO): measures how far the heterozygous cluster center sits above or below the homozygous cluster centers in the size dimension (y axis). Default ≥ −0.1. (c) Homozygous ratio offset (HomRO): measures the distance to zero in the contrast dimension (x axis) from the center of the homozygous cluster that is closest to zero. The minimum distance is between zero and BB, HomRO = 1.58. Default: ≥−0.9.

According to the analysis of 101 SNP markers recommended by ISAG in 93 horses, the HomeFLD ranged from 5.600 (MNEc_2_3_87970209_BIEC2_7996664) to 26.199 (MNEc_2_2_78264598_BIEC2_491394), with an average of 15.611. In turn, the HetSO averaged 0.369 and ranged from 0.000 (MNEc_2_3_87970209_BIEC2_799664) to 0.769 (MNEc_2_12_14391372_BIEC2_183251). Moreover, the HomeRO averaged 1.644 and ranged from 0.292 (MNEc_2_5_76618727_BIEC2_919835) to 3.056 (MNEc_2_2_2_78264598_BIEC2_491394). The MAF averaged 0.420 and ranged from 0.101 (MNEc_2_3_87970209_BIEC2_7996664) to 0.500 (MNEc_2_8_91677016_BIEC2_1066179). Finally, the Hardy–Weinberg equilibrium p-value averaged 0.398 and ranged from 0.000 (MNEc_2_4_78695209_BIEC2_870568) to 0.976 (MNEc_2_5_49304134_BIEC2_908630) (Table 1). As shown in Figure 1, FLD (10.09), HetSO (0.56), and HomRO (1.58) indicted a good SNP QC.

### Comparision of parentage testing using microsatellite markers and single nucleotide polymorphisms

The results of the paternity test using Ms markers and SNPs for each foal among Thoroughbred horses and Quarter horses are reported in Tables 2 and 3. Among the 15 Ms markers, AHT5, ASB2, ASB17, ASB23, CA425, HMS7, HTG10, and LEX3 did not obey Mendel’s genetic laws in Thoroughbred horses, whereas in Quarter horses, the AHT4, ASB2, and HMS2 markers alone were consistent with Mendel’s genetic law, therefore, paternity was not established.

In turn, among the 101 SNPs analyzed here, 31 SNPs (including MNEc_2_2_2_98568918_BIEC2_502451) in Thoroughbred horses, and 30 SNPs (including MNEc_2_30_7430735_BIEC2_816793) in Quarter horses were not established for parentage testing because of disobedience of Mendel’s genetic law.

## DISCUSSION

The objective of the present study was to construct a parentage-testing system for parentage testing of horse breeds (including Thoroughbred horses) using SNP markers, and to evaluate its utility for routine parentage testing by comparing it to the system used currently, which is based on Ms markers.

For over two decades, parentage control for horses in Korea has been carried out using the Ms parentage panel recommended by the ISAG (https://www.isag.us/) [13]. In Korea, 15 Ms markers are used to conduct parentage testing for approximately 2,000 Thoroughbred horses each year. Parentage verification is determined using Mendel’s genetic law. If a discrepancy is detected in one marker after the test judgment, it is assumed to be a mutation and additionally examined using three to five TKY (TKY279, TKY301, TKY312, TKY321, TKY341) markers; if two or more markers deviate from Mendel’s genetic law, it is finally established that paternity can be ruled out [1].

Although the use of Ms markers sometimes results in slippage mutation errors, these genotyping systems are widely used by horse registries worldwide. In turn, SNPs are biallelic sequences that are abundantly dispersed throughout most eukaryotic genomes [14–16] and exhibit the following characteristics: i) they have very low mutation rates compared with Ms markers (10 to 8 vs 10 to 3); ii) because of their short length they are highly suitable for analysis using automated high-throughput technologies; and iii) they can be detected using various techniques, such as TaqMan assays [17]. Therefore, there is a growing interest in the application of SNPs in the field of forensics [18,19]. Recently, the interest in the use of SNPs for parentage testing among domestic animals has increased [20,21]; moreover with the completion of the horse genome project, numerous SNPs have also become available for genetic studies in horses. One of the main proposed benefits of SNP-based systems is the potential for genetic diagnosis, such as the determination of coat color [22] and diseases, in horses, which are expected to help the actual coat color information and clinical symptoms gained by observation, because coat color information is particularly used currently for horse identification and registry. Therefore, this is a flexible technology that can be improved to serve further demands; for example, for the genotyping of factors such as coat color. However, there is insufficient information on their utility and related research to SNPs including parentage testing in horses in Korea. This study is the first discovery on horse parentage testing using SNPs; it shows the potential of SNPs testing as a basic tool for improving and developing the current system of horses parentage testing in Korea. This study confirmed that SNPs can be used in place of Ms markers for parentage testing in horses. However, further research using more samples is needed.

## Figures and Tables

**Table 1:** HomFLD, HetSO, HOmRO, MAF, H.W. p-value of 101 SNPs in 93 horses

SNPs ID	HomFLD	HetSO	HomRO	MAF	H.W.p-Value
MNEc_2_2_98568918_BIEC2_502451	15.170	0.431	1.855	0.359	0.941
MNEc_2_14_82654066_BIEC2_270795	16.772	0.338	1.722	0.414	0.979
MNEc_2_18_17553849_BIEC2_407206	16.637	0.475	1.676	0.382	0.282
MNEc_2_30_7430735_BIEC2_816793	19.578	0.263	1.435	0.409	0.130
MNEc_2_10_47218042_BIEC2_121102	11.735	0.199	1.007	0.363	0.169
MNEc_2_17_77806500_BIEC2-385477	12.522	0.254	1.317	0.468	0.491
MNEc_2_1_137298520_BIEC2-60186	15.537	0.185	1.418	0.430	0.237
MNEc_2_5_92328534_BIEC2_929536	12.251	0.200	0.798	0.387	0.398
MNEc_2_5_66442987_BIEC2_914714	10.674	0.180	1.128	0.478	0.044
MNEc_2_16_35399474_BIEC2_340595	15.971	0.241	1.101	0.457	0.809
MNEc_2_17_27133573_BIEC2_374571	22.094	0.398	2.153	0.473	0.364
MNEc_2_9_54440845_BIEC2_1095371	12.319	0.296	1.545	0.296	0.352
MNEc_2_5_96337109_BIEC2_931907	18.263	0.207	1.142	0.419	0.784
MNEc_2_1_87469882_BIEC2_37091	9.770	0.304	1.399	0.371	0.328
MNEc_2_5_57144900_BIEC2-910827	9.485	0.298	1.523	0.399	0.028
MNEc_2_17_17580290_BIEC2-369699	13.777	0.393	1.830	0.479	0.478
MNEc_2_3_89642978_BIEC2_800511	11.679	0.262	1.080	0.419	0.366
MNEc_2_16_40740308_BIEC2_342881	16.765	0.257	1.363	0.409	0.527
MNEc_2_10_58909591_BIEC2_126732	19.109	0.387	2.555	0.484	0.355
MNEc_2_6_31320852_BIEC2_946446	21.420	0.451	2.275	0.462	0.010
MNEc_2_16_81464884_BIEC2_364741	15.619	0.306	1.637	0.425	0.604
MNEc_2_10_43452669_BIEC2_119640	11.429	0.319	1.191	0.441	0.098
MNEc_2_25_26891173_BIEC2_667195	13.957	0.488	1.786	0.495	0.116
MNEc_2_4_78695209_BIEC2_870568	7.494	0.189	1.127	0.435	0.000
MNEc_2_29_28135792_BIEC2_761851	14.137	0.358	2.015	0.231	0.036
MNEc_2_31_17012751_BIEC2_839012	11.382	0.355	1.565	0.331	0.021
MNEc_2_23_20156067_BIEC2_618284	10.840	0.247	0.649	0.419	0.962
MNEc_2_11_7652121_BIEC2_136591	17.934	0.233	1.281	0.473	0.364
MNEc_2_3_87970209_BIEC2_799664	5.600	0.000	0.510	0.101	0.000
MNEc_2_3_99720005_BIEC2_806771	17.873	0.318	2.209	0.339	0.013
MNEc_2_8_53676953_BIEC2_1052417	10.951	0.285	1.659	0.445	0.390
MNEc_2_1_58021292_BIEC2_23891	16.422	0.157	1.232	0.333	0.087
MNEc_2_26_29137373_BIEC2_692543	15.810	0.233	1.385	0.419	0.784
MNEc_2_5_49304134_BIEC2_908630	19.368	0.454	2.158	0.360	0.976
MNEc_2_16_57363_BIEC2_326637	24.292	0.347	1.582	0.457	0.311
MNEc_2_8_61558651_BIEC2_1057053	18.911	0.412	2.154	0.366	0.483
MNEc_2_19_40010312_BIEC2_439060	11.687	0.253	0.861	0.434	0.429
MNEc_2_10_4630934_BIEC2_95522	19.251	0.415	1.995	0.419	0.880
MNEc_2_2_46721741_BIEC2_476920	13.343	0.374	1.704	0.489	0.348
MNEc_2_19_52162004_BIEC2_444978	10.628	0.243	1.260	0.473	0.512
MNEc_2_2_17760944_BIEC2_459311	8.571	0.158	1.279	0.442	0.652
MNEc_2_3_38344351_BIEC2_777914	21.125	0.503	2.409	0.446	0.759
MNEc_2_22_1966825_BIEC2_575436	8.459	0.067	0.498	0.472	0.893
MNEc_2_28_4835218_BIEC2_725322	12.867	0.374	1.060	0.473	0.857
MNEc_2_20_32691408_BIEC2_530788	23.791	0.249	1.850	0.312	0.644
MNEc_2_19_45732627_BIEC2_442892	16.697	0.298	1.768	0.446	0.840
MNEc_2_31_16284855_BIEC2_838630	11.257	0.223	1.374	0.500	0.211
MNEc_2_4_82173344_BIEC2_872142	11.376	0.337	1.585	0.317	0.432
MNEc_2_28_20911634_BIEC2_734103	19.514	0.405	1.960	0.419	0.784
MNEc_2_1_80029939_BIEC2_34987	13.715	0.356	2.318	0.473	0.734
MNEc_2_12_28692405_BIEC2_197740	22.518	0.393	2.030	0.414	0.650
MNEc_2_2_22843318_BIEC2_462271	17.686	0.583	2.584	0.457	0.627
MNEc_2_10_51447198_BIEC2_123002	24.488	0.429	2.777	0.344	0.170
MNEc_2_5_76618727_BIEC2_919835	7.451	0.167	0.292	0.333	0.453
MNEc_2_26_4719683_BIEC2_679734	11.106	0.235	1.279	0.288	0.026
MNEc_2_13_3048848_BIEC2_204153	17.683	0.498	2.056	0.446	0.291
MNEc_2_20_48021804_BIEC2_535766	13.078	0.348	1.924	0.393	0.769
MNEc_2_7_78027403_BIEC2_1007607	18.627	0.332	1.883	0.468	0.269
MNEc_2_24_17587744_BIEC2_636987	15.189	0.132	1.525	0.500	0.017
MNEc_2_16_70330928_BIEC2_358061	12.405	0.315	1.543	0.403	0.629
MNEc_2_23_4074195_BIEC2_606469	15.614	0.266	1.536	0.344	0.249
MNEc_2_16_7136293_BIEC2_328954	13.479	0.258	1.529	0.446	0.909
MNEc_2_2_78264598_BIEC2_491394	26.199	0.650	3.056	0.446	0.534
MNEc_2_4_729760_BIEC2-842401	22.706	0.442	1.822	0.462	0.377
MNEc_2_14_69974652_BIEC2_263616	7.228	0.207	0.911	0.451	0.304
MNEc_2_27_37890492_BIEC2_721061	24.313	0.327	1.515	0.500	0.756
MNEc_2_10_7585534_BIEC2_97679	11.683	0.232	0.864	0.489	0.121
MNEc_2_27_18605479_BIEC2_708018	24.322	0.468	2.066	0.441	0.975
MNEc_2_3_85958030_BIEC2-798927	21.235	0.256	2.710	0.473	0.031
MNEc_2_16_23357570_BIEC2-334065	23.937	0.428	2.463	0.398	0.001
MNEc_2_12_18434836_BIEC2_189666	16.885	0.413	1.810	0.489	0.005
MNEc_2_16_26521730_BIEC2-336269	11.333	0.124	0.979	0.467	0.040
MNEc_2_18_2187680_BIEC2-390920	15.179	0.205	1.267	0.500	0.048
MNEc_2_10_63490333_BIEC2_129063	10.575	0.147	0.751	0.385	0.117
MNEc_2_4_21403144_BIEC2_853347	12.733	0.448	1.862	0.430	0.008
MNEc_2_1_152166837_BIEC2_65145	15.696	0.526	2.276	0.376	0.212
MNEc_2_11_50291108_BIEC2_158202	24.886	0.436	2.821	0.419	0.047
MNEc_2_20_39580726_BIEC2_532106	16.943	0.538	2.246	0.468	0.269
MNEc_2_22_18630734_BIEC2_584911	20.870	0.465	2.845	0.479	0.903
MNEc_2_16_82667438_BIEC2_364945	9.743	0.197	1.033	0.466	0.259
MNEc_2_15_1250294_BIEC2_278532	15.132	0.367	1.495	0.306	0.269
MNEc_2_6_12344881_BIEC2_940642	25.731	0.518	2.174	0.398	0.755
MNEc_2_9_24877093_BIEC2_1080866	14.280	0.411	1.255	0.479	0.903
MNEc_2_3_117726644_BIEC2_811886	9.935	0.334	1.192	0.446	0.466
MNEc_2_28_29819475_BIEC2_737704	17.777	0.521	2.254	0.398	0.156
MNEc_2_1_79170604_BIEC2_34560	19.243	0.313	2.078	0.414	0.650
MNEc_2_14_15542132_BIEC2_245923	9.285	0.281	1.667	0.466	0.782
MNEc_2_15_51677625_BIEC2_310269	9.065	0.116	0.444	0.352	0.907
MNEc_2_11_56880157_BIEC2_162245	18.336	0.250	1.413	0.473	0.450
MNEc_2_8_90673387_BIEC2_1066033	13.017	0.300	1.714	0.446	0.466
MNEc_2_8_69858516_BIEC2_1061845	17.379	0.253	1.704	0.468	0.575
MNEc_2_4_17762202_BIEC2_851415	14.863	0.299	1.204	0.479	0.005
MNEc_2_18_81817905_BIEC2_421977	18.454	0.326	1.944	0.371	0.328
MNEc_2_18_34550094_BIEC2_410596	24.431	0.603	2.802	0.479	0.341
MNEc_2_18_56843481_BIEC2_415315	11.308	0.311	1.321	0.425	0.073
MNEc_2_8_91677016_BIEC2_1066179	17.302	0.388	1.387	0.500	0.178
MNEc_2_1_164591659_BIEC2_78523	18.608	0.454	2.300	0.468	0.052
MNEc_2_19_17235633_BIEC2_430577	13.034	0.224	1.245	0.393	0.564
MNEc_2_1_23891555_BIEC2_11336	27.580	0.748	2.571	0.430	0.351
MNEc_2_13_18106889_BIEC2_214346	11.585	0.281	1.406	0.382	0.130
MNEc_2_12_14391372_BIEC2_183251	8.787	0.769	1.850	0.335	0.075
Mean	15.611	0.335	1.644	0.420	0.398

FLD, fishers linear discriminant; HetSO, heterozygous strength offset; HomRO, homozygous ratio offset; MAF, minorallelefrequency; H.W. p-value, Hardy–Weinberg equilibrium p-value; SNP, single nucleotide polymorphisms.

**Table 2:** A case of exclusion in horse parentage test using microsatellite markers

Items	Ms loci															Breeds

	AHT4	AHT5	ASB2	HMS3	HMS6	HMS7	HTG4	HTG10	VHL20	ASB17	ASB23	HMS1	LEX3	CA425	HMS2	
Sire	H/O	K/M	M/Q	I/O	P/P	M/N	K/M	I/I	L/M	N/O	K/K	J/M	M/-	N/O	K/L	TB
Dam	J/O	J/K	N/R	I/O	P/P	O/O	K/K	I/R	I/I	N/R	J/S	J/M	H/O	J/N	K/L	
Foal	O/O	**M/N**	**M/Q**	I/I	P/P	**O/O**	K/M	**I/L**	I/L	**G/G**	**J/L**	J/M	**N/-**	**J/M**	L/L	
Sire	I/O	J/N	K/M	I/N	L/P	N/O	P/P	L/S	I/M	M/Q	J/U	M/M	O/O	J/M	M/O	QH
Dam	H/O	N/N	K/K	I/I	L/O	O/O	K/P	R/S	I/Q	N/Q	L/U	J/M	L/O	J/N	J/O	
Foal	O/O	**J/O**	K/K	**N/R**	**P/P**	**J/J**	**K/M**	**L/O**	**M/O**	**N/R**	**S/U**	**J/J**	**L/L**	**N/O**	O/O	

Red color indicates a marker that is inconsistent with Mendel’s genetic laws.

**Table 3:** A case of exclusion in horse parentage test using SNPs

SNPs ID	TB			QH		
	
	Sire	Dam	Foal	Sire	Dam	Foal
MNEc_2_2_98568918_BIEC2_502451	AB	AA	BB	AB	AB	AB
MNEc_2_14_82654066_BIEC2_270795	AA	BB	AB	AB	AB	AA
MNEc_2_18_17553849_BIEC2_407206	AA	AA	BB	BB	BB	BB
MNEc_2_30_7430735_BIEC2_816793	AB	AB	AB	AB	BB	AA
MNEc_2_10_47218042_BIEC2_121102	AA	AA	AB	AA	AB	AA
MNEc_2_17_77806500_BIEC2-385477	AB	AB	AB	AB	AB	AA
MNEc_2_1_137298520_BIEC2-60186	AB	AB	AB	AB	BB	AB
MNEc_2_5_92328534_BIEC2_929536	AB	BB	AB	AA	AA	AB
MNEc_2_5_66442987_BIEC2_914714	AB	AB	AB	AA	AA	AB
MNEc_2_16_35399474_BIEC2_340595	AB	AB	AB	AA	AB	AA
MNEc_2_17_27133573_BIEC2_374571	AA	AB	AA	AA	AA	BB
MNEc_2_9_54440845_BIEC2_1095371	BB	BB	BB	AB	AB	AB
MNEc_2_5_96337109_BIEC2_931907	AA	AB	AA	AA	AB	AA
MNEc_2_1_87469882_BIEC2_37091	AB	AA	AA	AA	AB	BB
MNEc_2_5_57144900_BIEC2-910827	AA	AB	AB	AB	AB	AA
MNEc_2_17_17580290_BIEC2-369699	BB	AB	AB	BB	BB	AB
MNEc_2_3_89642978_BIEC2_800511	BB	AB	AB	AB	AB	BB
MNEc_2_16_40740308_BIEC2_342881	AB	AB	AB	BB	BB	AA
MNEc_2_10_58909591_BIEC2_126732	AB	BB	AA	AB	AB	AA
MNEc_2_6_31320852_BIEC2_946446	AB	AA	AA	AB	BB	AB
MNEc_2_16_81464884_BIEC2_364741	BB	BB	AB	AA	AA	AB
MNEc_2_10_43452669_BIEC2_119640	BB	AA	AA	AB	AB	AA
MNEc_2_25_26891173_BIEC2_667195	AA	AA	AB	AA	AA	AA
MNEc_2_4_78695209_BIEC2_870568	NoCall	AA	AA	AB	AA	AA
MNEc_2_29_28135792_BIEC2_761851	AA	AA	AB	AB	AB	AA
MNEc_2_31_17012751_BIEC2_839012	AA	AA	AA	AB	AB	BB
MNEc_2_23_20156067_BIEC2_618284	AB	BB	BB	BB	AB	BB
MNEc_2_11_7652121_BIEC2_136591	BB	AA	AA	AA	AB	AA
MNEc_2_3_87970209_BIEC2_799664	BB	BB	BB	BB	BB	BB
MNEc_2_3_99720005_BIEC2_806771	BB	AB	AA	BB	AB	AB
MNEc_2_8_53676953_BIEC2_1052417	BB	AB	AB	AB	AB	AB
MNEc_2_1_58021292_BIEC2_23891	AA	AA	AA	AB	AB	AA
MNEc_2_26_29137373_BIEC2_692543	AB	BB	AB	AB	BB	AA
MNEc_2_5_49304134_BIEC2_908630	BB	AB	AB	BB	AB	BB
MNEc_2_16_57363_BIEC2_326637	AA	AB	AB	BB	AB	AA
MNEc_2_8_61558651_BIEC2_1057053	AB	BB	AA	AB	AB	BB
MNEc_2_19_40010312_BIEC2_439060	AB	AA	AA	AA	AA	BB
MNEc_2_10_4630934_BIEC2_95522	AB	AB	AB	AB	AB	AB
MNEc_2_2_46721741_BIEC2_476920	BB	AB	AB	BB	BB	AB
MNEc_2_19_52162004_BIEC2_444978	AA	AB	AB	AB	AB	BB
MNEc_2_2_17760944_BIEC2_459311	AB	AA	AB	AA	AB	AA
MNEc_2_3_38344351_BIEC2_777914	AB	AA	AA	AB	AB	NoCall
MNEc_2_22_1966825_BIEC2_575436	AB	BB	AB	AA	AA	NoCall
MNEc_2_28_4835218_BIEC2_725322	AA	AB	AB	BB	AB	AB
MNEc_2_20_32691408_BIEC2_530788	BB	AA	AB	AB	BB	AB
MNEc_2_19_45732627_BIEC2_442892	BB	BB	BB	BB	BB	AB
MNEc_2_31_16284855_BIEC2_838630	AB	AB	AA	AB	AB	AB
MNEc_2_4_82173344_BIEC2_872142	AA	AA	AB	AA	AA	AB
MNEc_2_28_20911634_BIEC2_734103	AB	AB	AA	AA	AA	AB
MNEc_2_1_80029939_BIEC2_34987	AA	AB	AA	AB	AB	AB
MNEc_2_12_28692405_BIEC2_197740	BB	BB	BB	AB	BB	AB
MNEc_2_2_22843318_BIEC2_462271	AA	AB	AA	AA	AA	AA
MNEc_2_10_51447198_BIEC2_123002	AA	AA	AB	BB	BB	AA
MNEc_2_5_76618727_BIEC2_919835	AB	NoCall	AB	BB	AB	BB
MNEc_2_26_4719683_BIEC2_679734	BB	BB	BB	AA	AB	AA
MNEc_2_13_3048848_BIEC2_204153	AB	AA	AB	AB	BB	BB
MNEc_2_20_48021804_BIEC2_535766	AB	BB	BB	AB	BB	AB
MNEc_2_7_78027403_BIEC2_1007607	BB	AA	BB	BB	AB	AB
MNEc_2_24_17587744_BIEC2_636987	BB	BB	AB	AA	AA	AA
MNEc_2_16_70330928_BIEC2_358061	AA	AB	BB	AB	BB	AA
MNEc_2_23_4074195_BIEC2_606469	AB	BB	BB	BB	AB	AB
MNEc_2_16_7136293_BIEC2_328954	AB	AB	AA	AA	AA	AB
MNEc_2_2_78264598_BIEC2_491394	BB	AB	BB	BB	AB	AA
MNEc_2_4_729760_BIEC2-842401	AB	BB	AA	AB	AB	AA
MNEc_2_14_69974652_BIEC2_263616	AB	AA	NoCall	AB	AA	AA
MNEc_2_27_37890492_BIEC2_721061	BB	AB	BB	AA	AB	BB
MNEc_2_10_7585534_BIEC2_97679	BB	AB	BB	AB	AA	BB
MNEc_2_27_18605479_BIEC2_708018	AB	AB	AB	AB	AB	AB
MNEc_2_3_85958030_BIEC2-798927	AA	AB	AA	BB	BB	BB
MNEc_2_16_23357570_BIEC2-334065	AB	AB	AA	BB	BB	AB
MNEc_2_12_18434836_BIEC2_189666	BB	AA	AA	AB	AB	AA
MNEc_2_16_26521730_BIEC2-336269	AA	AA	AB	AB	AB	AB
MNEc_2_18_2187680_BIEC2-390920	AA	AA	AA	AB	AB	AB
MNEc_2_10_63490333_BIEC2_129063	BB	BB	BB	AB	BB	AB
MNEc_2_4_21403144_BIEC2_853347	BB	BB	BB	AB	AA	AB
MNEc_2_1_152166837_BIEC2_65145	BB	AA	BB	BB	BB	AB
MNEc_2_11_50291108_BIEC2_158202	AA	AA	AB	BB	BB	AB
MNEc_2_20_39580726_BIEC2_532106	AA	AB	AA	AB	BB	AB
MNEc_2_22_18630734_BIEC2_584911	AB	AA	AB	AB	AB	AB
MNEc_2_16_82667438_BIEC2_364945	AB	BB	AB	BB	AB	BB
MNEc_2_15_1250294_BIEC2_278532	AA	BB	AB	AA	AB	AA
MNEc_2_6_12344881_BIEC2_940642	AB	AA	BB	AB	AB	AB
MNEc_2_9_24877093_BIEC2_1080866	BB	BB	AB	BB	BB	BB
MNEc_2_3_117726644_BIEC2_811886	AB	AB	AB	AB	BB	AB
MNEc_2_28_29819475_BIEC2_737704	AB	AA	AB	AB	AA	BB
MNEc_2_1_79170604_BIEC2_34560	AA	BB	AA	AB	AA	BB
MNEc_2_14_15542132_BIEC2_245923	BB	BB	AA	AB	NoCall	AA
MNEc_2_15_51677625_BIEC2_310269	AA	AB	BB	AB	BB	AB
MNEc_2_11_56880157_BIEC2_162245	AA	AA	BB	AB	AB	AA
MNEc_2_8_90673387_BIEC2_1066033	AA	AA	AB	AB	AB	AB
MNEc_2_8_69858516_BIEC2_1061845	AB	AB	AA	BB	AB	AB
MNEc_2_4_17762202_BIEC2_851415	AA	AA	AA	AB	BB	AB
MNEc_2_18_81817905_BIEC2_421977	AB	AA	AA	AA	AB	AA
MNEc_2_18_34550094_BIEC2_410596	AA	AA	AA	AA	AA	AB
MNEc_2_18_56843481_BIEC2_415315	BB	AB	AA	AA	AB	AA
MNEc_2_8_91677016_BIEC2_1066179	BB	AB	AA	AB	AB	AB
MNEc_2_1_164591659_BIEC2_78523	BB	AA	BB	AB	AB	AA
MNEc_2_19_17235633_BIEC2_430577	BB	BB	AB	AA	AA	AA
MNEc_2_1_23891555_BIEC2_11336	BB	BB	BB	AB	AB	BB
MNEc_2_13_18106889_BIEC2_214346	AB	AB	BB	AA	AA	BB
MNEc_2_12_14391372_BIEC2_183251	BB	BB	BB	AB	AB	BB

SNP, single nucleotide polymorphisms.

* SNP bases A and T were classified as allele A, and C and G were classified as allele B.
